# Analysis of the Chaotic Component of Photoplethysmography and Its Association with Hemodynamic Parameters

**DOI:** 10.3390/e25121582

**Published:** 2023-11-24

**Authors:** Xiaoman Xing, Wen-Fei Dong, Renjie Xiao, Mingxuan Song, Chenyu Jiang

**Affiliations:** 1 School of Biomedical Engineering (Suzhou), Division of Life Sciences and Medicine, University of Sciences and Technology of China, Suzhou 215163, China; 2 Suzhou Institute of Biomedical Engineering and Technology, Chinese Academy of Sciences, Suzhou 215163, China; 3 Medical Health Information Center, Suzhou Institute of Biomedical Engineering and Technology, Chinese Academy of Sciences, Suzhou 215163, China; 4 Suzhou GK Medtech Science and Technology Development (Group) Co., Ltd., Suzhou 215163, China; 5 Jinan Guoke Medical Technology Development Co., Ltd., Jinan 250100, China

**Keywords:** photoplethysmography, chaotic analysis, fractal dimension, hemodynamic parameters

## Abstract

Wearable technologies face challenges due to signal instability, hindering their usage. Thus, it is crucial to comprehend the connection between dynamic patterns in photoplethysmography (PPG) signals and cardiovascular health. In our study, we collected 401 multimodal recordings from two public databases, evaluating hemodynamic conditions like blood pressure (BP), cardiac output (CO), vascular compliance (*C*), and peripheral resistance (*R*). Using irregular-resampling auto-spectral analysis (IRASA), we quantified chaotic components in PPG signals and employed different methods to measure the fractal dimension (FD) and entropy. Our findings revealed that in surgery patients, the power of chaotic components increased with vascular stiffness. As the intensity of CO fluctuations increased, there was a notable strengthening in the correlation between most complexity measures of PPG and these parameters. Interestingly, some conventional morphological features displayed a significant decrease in correlation, indicating a shift from a static to dynamic scenario. Healthy subjects exhibited a higher percentage of chaotic components, and the correlation between complexity measures and hemodynamics in this group tended to be more pronounced. Causal analysis showed that hemodynamic fluctuations are main influencers for FD changes, with observed feedback in most cases. In conclusion, understanding chaotic patterns in PPG signals is vital for assessing cardiovascular health, especially in individuals with unstable hemodynamics or during ambulatory testing. These insights can help overcome the challenges faced by wearable technologies and enhance their usage in real-world scenarios.

## 1. Introduction

Ambulatory cardiovascular function monitoring plays a vital role in the timely diagnosis and management of cardiovascular diseases. Wearable devices offer a convenient approach to monitoring essential cardiovascular parameters, including heart rate (HR), respiratory rate (RR), cardiac output (CO), and blood pressure (BP), utilizing signals such as photoplethysmography (PPG), ballistocardiogram (BCG), and electrocardiogram (ECG) [1,2,3]. While accurate calculations of HR and RR are attainable, achieving a higher accuracy in monitoring cardiovascular functionality through noninvasive wearable methods remains a challenge. Advanced techniques, such as long- and short-term memory neural networks (LSTM) and transformer neural networks, show promise in capturing the complex and nonlinear relationships between measured signals and neighboring data [2,4]. However, the precise nature of these time series interactions is not yet fully understood.

The utilization of fractal and entropy analyses provides valuable insights into the relationship between cardiovascular function and the temporal complexities of the associated signals. For example, Christ et al. conducted a study where they found that the Hausdorff fractal dimension could be used to monitor hemodynamics during an elective aortic aneurysm repair. They observed a higher fractal dimension in the PPG signal during aortic clamping and declamping (*p* = 0.08) [5]. Khodabakhshi et al. explored the monitoring of BP using fractal dimension, the Lyapunov exponent, and recurrence quantification analysis (RQA) [6]. Their findings indicated that incorporating chaotic and other temporal features enhance deep-learning performance in estimating BP from PPG. Prabhakar et al. employed fuzzy entropy and approximate entropy (ApEn) to stratify the cardiovascular risk [7]. Similarly, McManus et al. utilized the Shannon Entropy and Poincare plots for atrial fibrillation (AF) detection [8]. Chen et al. and Rantanen et al. identified various entropy measures, including ApEn, sample entropy (SampEn), response entropy, and state entropy, which demonstrated correlations with analgesia depth [9,10]. Wei et al. found that the percussion entropy index (PEI) serves as a significant risk parameter for newly onset diabetic peripheral neuropathy (DPN) [11].

Heart rate variability (HRV) is also a well-studied index that demonstrates a strong association with hemodynamics [12,13,14]. Nevertheless, the calculation of HRV is typically based on ECG, while limited attention has been given to investigating the connection between complexities of PPG, specifically its fractal dimension (FD), and hemodynamics. 

In recent studies conducted by Sviridova et al., it was found that PPG signals exhibit characteristics of chaotic motion resembling Rössler’s single band chaos [15,16]. They also proposed that the chaotic components observed in PPG signals might be generated by variations in blood volume [16]. However, the significance of PPG fractal components in relation to factors, such as age, BP, and hemodynamic variation, remains uncertain. The influence of these factors on the chaotic behavior of PPG signals has yet to be established. Therefore, this paper aims to quantify the fractal components present in PPG signals and establish a causal relationship between these components and underlying hemodynamic changes. Investigating the interplay of these parameters will allow the development of reliable tools to enhance wearable hemodynamic estimation accuracy and monitor cardiovascular health. While we specifically employed PPG signals retrieved from publicly available databases as an illustrative example, it is important to note that other technologies, such as BCG, can also benefit from similar mechanism studies.

To build a link between PPG complexities and hemodynamics, we employed the classical four-element Windkessel (WK4) model [17,18]. Interestingly, the equations describing this model bear resemblance to the Rössler system, which aligns with the findings reported by Sviridova et al. In the context of an unstable state in the cardiovascular closed-loop system, our hypothesis suggests that there is a significant exchange of energy within the temporal patterns. This phenomenon may be attributed to hysteresis of vascular responses and variations in active cardiac activities. As a result, complexity features may become more pronounced.

This paper is organized as follows. First, we present the publicly available database and describe the data standardization procedure employed to validate our hypothesis. Second, we provide a concise introduction to the WK4 model and model-based hemodynamic estimation, which serves to provide a plausible physiological explanation. Next, we quantify the chaotic components and compute several widely used fractal and entropy measures, including HRV, to assess their responsiveness to the hemodynamic state and the variation in sensitivity due to fluctuations in hemodynamics. Subsequently, we investigate the causal relationship between the FD, HRV, and hemodynamic parameters across a diverse range of hemodynamic conditions. By delving into potential physiological connections, our objective is to gain insight into the underlying mechanisms and implications associated with these findings.

## 2. Materials and Methods

### 2.1. Data Collection and Preprosessing

In this study, we employed two databases to establish a relationship between complexity measures and hemodynamics. The first database we utilized is VitalDB, which is a comprehensive database specifically designed for storing and managing time series data of vital signs in surgery patients and patients with ICU stays [19]. The database encompasses a wide range of vital sign parameters, including hemodynamic measurements, blood gas values, and ventilator settings. The second database, maintained by Carlson et al., consists of data from 40 healthy subjects. This database provides a multimodal approach to capturing vital sign data [20]. Each subject underwent measurements in a supine position for an approximate duration of 5 min.

**Figure 1:**
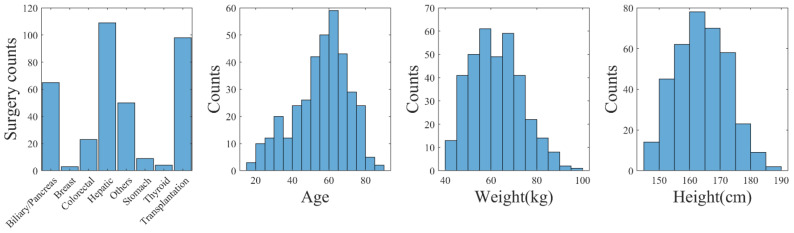
Distribution of biometrics and surgery types (VitalDB).

Considering the significant variability of data in real clinical settings, we implemented measures to ensure relative consistency. Specifically, we limited the age range of the subjects to those over 18 years old, with a weight greater than 40 kg, a height greater than 145 cm, and a body mass index between 16 and 35. Furthermore, measurements obtained in lateral positions as well as suspected excessive blood loss (SBP < 80 mmHg) were excluded from the analysis. For this study, we included 361 patients who underwent various surgical procedures, consisting of 213 male and 148 female subjects. Vital signals, such as BP, PPG, and CO, were collected from these patients at a sampling rate of 50 Hz. Importantly, none of the patients included in this study had undergone vascular or heart surgeries. We also collected data from 40 healthy subjects, consisting of 17 males and 23 females, with similar physiological measurements. For a comprehensive overview of participants’ information, please refer to Figure 1 and Table 1. We also presented the 1st percentile (1%) and 99th percentile (99%) for each parameter to establish the boundaries of the data. Outliers in systolic blood pressure (SBP) and diastolic blood pressure (DBP) were identified as measurements outside this range. These outliers were then removed to minimize the potential impact of erroneous measurements.

The data length varied among patients, ranging from 2 to 6 h, depending on the duration of their surgical procedures. For data preprocessing, we used the algorithm proposed by Shin et al. [21] to detect the foot points of the PPG, which were then connected by a straight line and subtracted. We defined the baseline as the DC component of the PPG and the peak-to-peak amplitude of the detrended PPG as AC. Then, the data were subjected to data cleaning, wherein highly noisy and distorted data were discarded, and suitable time series were chosen [22,23].

**Figure 2:**
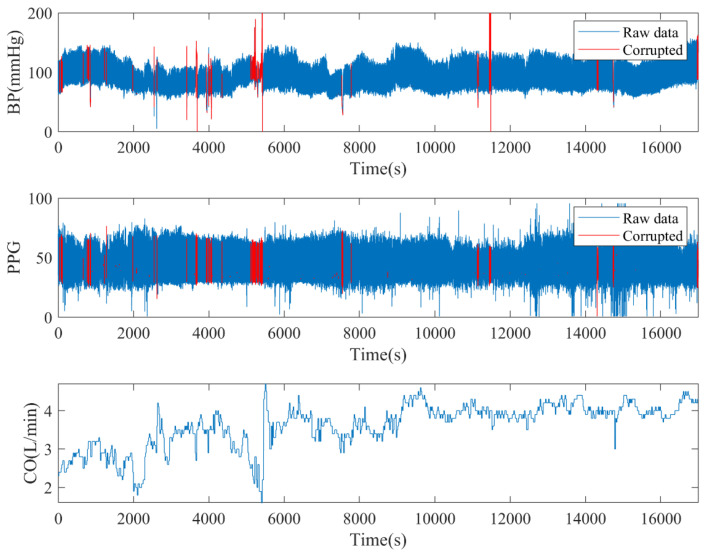
An illustration of multichannel signals from a subject in VitalDB with corruption detection.

To showcase the contents of the database, we chose an example and depicted the corresponding signals in Figure 2. Outlier detection was performed on a per cardiac cycle basis, employing the following criteria:

- Pulse pressure was smaller than 15 mmHg.
- The correlation coefficient of BP and the PPG waveform was less than 0.8 [24].
- Peak-to-peak distance of one “cardiac cycle” exceeded 1.5 s or was less than 0.5 s.
- PPG signal quality was considered unusable if the skewness of the PPG signal in any window was less than 0, as suggested by [25,26].

To conduct the fractal analysis on the raw signal, a sliding window of 100 s with a 5 s overlap was applied. On the other hand, for the fractal analysis of the per-beat signal amplitude, a sliding window of 100 cardiac cycles was utilized. If more than 10 s of signal was corrupt or labelled as outliers, the entire window was discarded.

### 2.2. Brief Introduction of the WK4 Model

**Figure 3:**
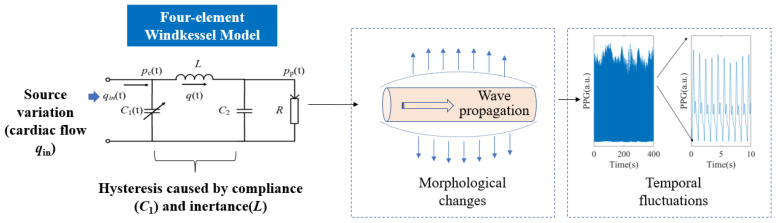
The WK4 model. Morphological features of PPG were generated by instantaneous vascular expansion and contraction. The temporal patterns were formed by the delayed vascular responses to varying *q*_in_. *p*_c_(t): blood flow in central vasculature.

PPG signals were generated by a classical WK4 model, as depicted in Figure 3. In this model, the heart was represented as a current source *q*_in_, which is closely related to the stroke volume (SV) and CO. The arterial tree system was modeled by four major parameters. *C*_1_ is the compliance of the major blood vessels [27]. *R* reflects the peripheral resistance [28]. The compliance of the distal arteries (*C*_2_) and inertance (*L*) were added to increase the PPG waveform fitting accuracy [29]. The peripheral BP (*p*_p_) was estimated with known cardiovascular and hemodynamic parameters. Time dependence was introduced to *C*_1_, *q*_in_, *p*_c_, and *p*_p_, indicating that these variables exhibit rapid fluctuations within a cardiac cycle. In contrast, other parameters may exhibit significantly slower rates of change.

The equations describing this system bear resemblance to the Rössler system, a well-known example capable of generating deterministic chaos.

(1) $$\left\{\begin{array}{c} \frac{dq\left(t\right)}{dt}=\frac{1}{L}\left(p_{c}\left(t\right)-p_{p}\left(t\right)\right)\\ \frac{dp_{c}\left(t\right)}{dt}=\frac{1}{C_{1}\left(t\right)}\left(q_{in}\left(t\right)-q\left(t\right)\right)\\ \frac{dp_{p}\left(t\right)}{dt}=\frac{1}{C_{2}}\left(q\left(t\right)-\frac{p_{p}\left(t\right)}{R}\right) \end{array}\right.$$

The WK4 model is a simplified 0D representation of the complex cardiovascular system. Its accuracy for quantification is limited. However, the model is straightforward and easily comprehensible. In this study, we aim to explain the experimental findings based on the existing physiological models.

### 2.3. Hemodynamic Parameter Estimation

The WK4 model serves as a physiological basis for estimating hemodynamic parameters. In order to streamline the estimation procedure, we adopted an alternative approach that initially estimated *C*_1_ and *R*. Subsequently, the WK4 model was employed to estimate *C*_2_ and *L*.

On a per-heartbeat basis, *R* was estimated by calculating the ratio of the mean arterial pressure (MAP) and CO [28]. The major compliance *C*_1_ was estimated by calculating the ratio of the peak-to-peak PPG amplitude and the pulse pressure (PP) of BP [30]. This simplified approach does not offer a precise estimation of *C*_1_. Nevertheless, in situations where a quick evaluation of vascular function is required, especially when relying solely on *C*_1_ to estimate the intrasubject correlation between hemodynamics and PPG features, a highly simplified method was adequate [30]. Peripheral compliance *C*_2_ and inertance *L* were estimated according to the WK4 model by decomposing blood flow into a Fourier series [31,32]. To optimize the model, values for *L* and *C*_2_ were selected to minimize the root–mean–square difference (RMS) between the model’s output *p*_p_ and the observed data. The RMS value was calculated as the square root of the sum of squared errors (SSE) divided by the degree of freedom N-1, following the formula $RMS=\sqrt{SSE/(N-1)}$. The detailed process followed the method proposed by Segers et al. [32].

### 2.4. Morphological Features of PPG

Morphological features derived from photoplethysmography (PPG) signals have been used to estimate hemodynamics. While the direct correlation coefficient may not be high, a combination of these features allows for a decent estimation of hemodynamic parameters, such as *C*_1_, BP, and CO. These features mainly capture the instantaneous response of the cardiovascular system to fluctuations in hemodynamics. In this study, we employed commonly used morphological features (as listed in Table 2) to compare with complexity measures.

### 2.5. Complexity Measures of PPG Signals

#### 2.5.1. Separating Fractal and Oscillatory Components

To begin, it was crucial to understand the significance of chaotic signals. It was only when the chaotic signal proved to be increasingly valuable that it became worth considering in our analysis.

**Figure 4:**
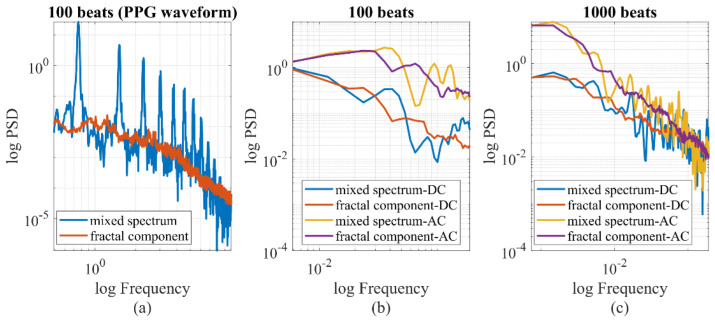
IRASA of PPG signals with different signal lengths and sampling rates. (**a**) At 50 Hz, the PPG signal is a mixture of oscillatory and chaotic components (0.5–10 Hz). (**b**,**c**) If the pulsatile amplitude data is sampled per heartbeat, the time series pattern of AC and DC is chaotic, with minimal oscillatory components.

To separate fractal and oscillatory components in the PPG signal, we employed the irregular-resampling auto-spectral analysis (IRASA) technique [38]. The IRASA method addresses limitations of the straightforward power spectrum method when prominent oscillations may deviate the power spectral density (PSD) from a power-law distribution [39]. It uses a resampling technique with multiple noninteger pairwise factors, including positive numbers and their reciprocals. By calculating the geometric mean of the auto-power spectra for each pair of resampled signals, the power associated with the oscillatory component was redistributed away from its original frequencies (fundamental and harmonic) by a frequency offset that varies with the resampling factor. In contrast, the power attributed to the fractal component maintains a consistent power-law statistical distribution, regardless of the resampling factor. An example of the IRASA is demonstrated in Figure 4. The analysis included two different signal lengths, 100 and 1000 heartbeats. Specifically, for the 100-heartbeat segment, both the IRASA of the PPG waveform at 50 Hz and the beat-to-beat baseline (DC) and pulsatile amplitude (AC) of the PPG were conducted [24]. The chaotic components exhibited higher noise levels and deviated from linearity at lower frequencies, as shown in Figure 4a. This deviation was likely due to multifractality at different timescales [38]. Figure 4b exhibited a higher level of noise compared to Figure 4c. With a longer recording time and lower sampling rate, minimal oscillation was observed in Figure 4c, suggesting that the long-term chaotic components are the main contributor to the power and require a longer duration to stabilize.

**Figure 5:**
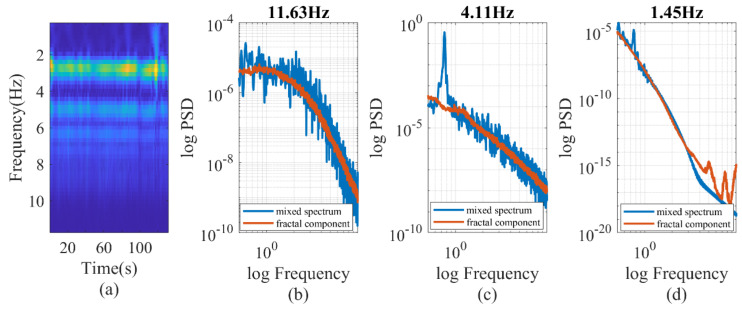
(**a**) wavelet decomposition of PPG signal. (**b**–**d**) IRASA of PPG signals at different wavelet frequencies.

To test the spectral difference, we decomposed the PPG signal into various wavelet components and individually analyzed each component to calculate its chaotic properties, as shown in Figure 5. For our investigation, we utilized the Morse wavelet and focused on wavelet components ranging from 0.4 Hz to 11.5 Hz. The frequency of each wavelet was established based on the sampling rate and the length of the signal. In this case, a consistent duration of 100 s was employed for all calculations, resulting in the scalogram having uniform frequency ticks. The wavelet component, centered around 4 Hz, showed a standard blend of oscillatory and chaotic behavior. Conversely, the wavelet component with higher frequencies (11.63 Hz) exhibited a behavior similar to chaos, although the spectrum deviated from linearity at lower spectral frequencies. On the other hand, the wavelet component with lower frequencies (1.45 Hz) mainly exhibited chaotic behavior, with a deviation from linearity at higher spectral frequencies. This observation suggests the presence of multifractal behavior at the higher spectral frequencies. The fractal analysis of the PPG raw signal resulted in overlapping individual spectral response patterns. The sum of the individual PSD of wavelets matched the oscillatory peaks and the shape of the PSD of the PPG raw signal. However, due to the potential nonorthogonality of the continuous wavelet decomposition, it is not advisable to numerically represent the PSD of the PPG raw signal by simply summing the wavelet PSDs.

#### 2.5.2. Calculation of Fractal Dimensions

Since the PPG signal was chaotic, we used fractal dimensions to describe its temporal patterns. The Higuchi fractal dimension (HFD) and Katz fractal dimension (KFD) are two of the most commonly used FD measures for physiological and neurological data [40]. The HFD, developed by Yoshimi Higuchi et al., is a measure that describes the degree of self-similarity of a time series signal [41]. It is computed by analyzing the fluctuations in the time series over different scales or window sizes and serves as a function of the curve length in relation to the granularity of the analysis scale. On the other hand, the KFD is computed by measuring the length of the curve as a function of the level of detail, which captures the overall complexity of the structure without considering its orientation [42].

The method for calculating the HFD has been previously described in the literature. In this study, we employed three different sampling approaches to determine the optimal correlation with hemodynamics: raw PPG waveform sampled at 50 Hz, wavelet spectral components sampled at 50 Hz, and beat-to-beat sampling of PPG–AC or PPG–DC. The wavelet decomposition resulted in a multiscale FD. For all three schemes, the maximum number of segments (k_max_) was set to 10.

The detailed calculation of the KFD was proposed by Katz et al. [42]. While the HFD provides more accurate estimations of fractal dimensions, some studies have shown that the KFD exhibits higher discriminative power in specific circumstances [40,43].

#### 2.5.3. Entropy Measures

**Figure 6:**
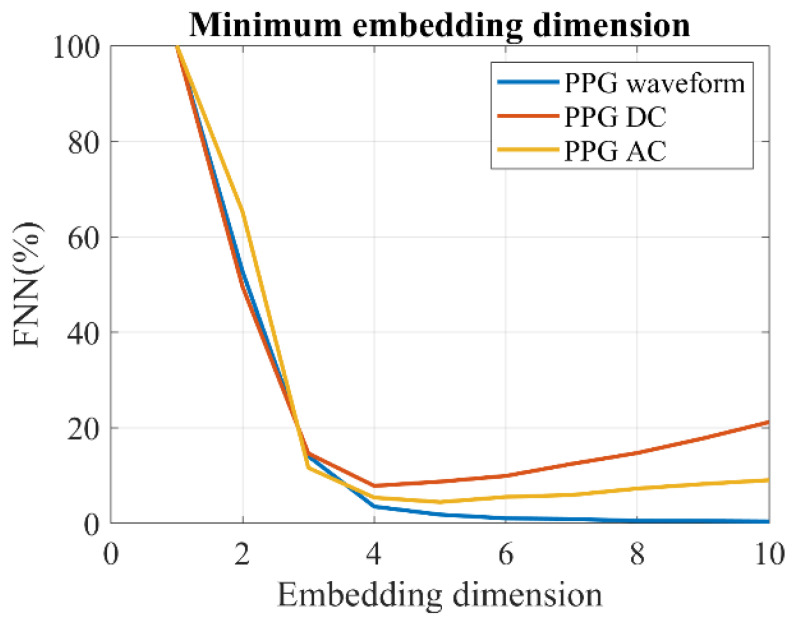
Estimation of the minimum embedding dimension of PPG by the FNN method.

To compare with the fractal analysis, we tested several popular entropy measures. The same three sampling approaches described in Section 2.5.2 were utilized. In order to improve the precision of the entropy estimation, we first calculated the embedded dimension. The embedded dimension refers to the number of variables or dimensions required to represent a complex or high-dimensional dataset in a lower-dimensional space. In our study, we employed the false nearest neighbors (FNN) method [44], as shown in Figure 6, to determine the embedded dimension. Based on the FNN calculation, we set the embedded dimension as four. It is worth noting that the decrease in the FNN of the PPG–AC and PPG–DC was not consistently monotonic, which may be attributed to the uneven sampling time of the PPG–DC and PPG–AC.

For the purpose of hemodynamic monitoring, we chose the following four sets of entropy measures to compare with the fractal dimensions:

- Sample Entropy (SampEn): SampEn was used to quantify the irregularity or unpredictability of the time series data. It assessed the complexity of a signal by measuring the likelihood of finding repeated patterns within a specified length [45].
- Approximate Entropy (ApEn): Similar to SampEn, ApEn measured the amount of unpredictability in the time series data, with higher values indicating greater complexity [46].
- Fuzzy Entropy (FuzzEn): FuzzEn is a measure of complexity that incorporates uncertainty or fuzziness in data by using fuzzy sets. It quantified the disorder or complexity in systems with imprecise or uncertain information [47].
- Recurrence quantification analysis (RQA): RQA measured properties, such as frequency, duration, and clustering of the recurrences, providing insights into the complexity and predictability of a system. In this study, we used entropy (ENTR) in RQA, due to its high relevance [48,49].

Heart rate variability (HRV) was also calculated as a comparison. HRV reflects the regulation of the autonomic nervous system and has been used to assess the risk of cardiovascular disease, among other conditions. We evaluated the LF/HF of the HRV. Here, the LF/HF (low frequency to high frequency ratio) is a measure of HRV that indicates the balance between sympathetic and parasympathetic nervous system activity [14]. A higher LF/HF ratio suggests greater sympathetic activity, while a lower ratio indicates more parasympathetic influence. The LF/HF is robust to occasional signal corruptions.

### 2.6. Causal Relationship between Hemodynamics and Complexity Measures

Our hypothesis suggests that variations in hemodynamics contribute to an increase in complexity. To investigate this, we computed the percentage power of chaotic components and compared it to hemodynamics and their fluctuations. Additionally, we hypothesized that the relevance of complexity measures would be magnified during unstable hemodynamic conditions. To explore this further, we calculated the intrasubject Pearson correlation coefficient to evaluate the sensitivity of complexity to hemodynamic status, taking into account the magnitude of hemodynamic fluctuations. This analysis was performed under different hemodynamic conditions and fluctuation levels, enabling meaningful comparisons to be made.

We also used the Granger causality test to investigate the relationship between hemodynamics and fractal patterns. Although the Granger causality does not establish true causation, it suggests the presence of a predictive relationship between variables. Our previous work demonstrated that hemodynamic fluctuation, including CO, *C*_1_, and *R*, contributes to the hysteresis of cardiovascular responses, leading to the formation of fractal patterns [18]. Along with the Granger causality test, our results provided supportive evidence for future, more rigorous investigations in this area. In this study, the significance level of the Granger test was set to 0.05, and the maximum lag time was set to be 50 s [50].

### 2.7. Statistical Tests

The intrasubject correlation coefficients (*r*) of features and hemodynamics were expected to follow a student’s t-distribution, which is robust when the sample size is large. In our study, each subject had an average recording time of 13,258 s, corresponding to approximately 3014 data points. We used a *p*-value of 0.05 to determine the statistical significance in the analysis.

To enable comparisons, we calculated the feature–hemodynamics correlation coefficient for each subject. The distribution of these complexity measures closely resembled a t-distribution, as determined by a student’s *t*-test (e.g., *p* = 0.12 for HFD). Therefore, to minimize potential biases associated with calculating mean values, we chose to utilize the median and standard deviation for intersubject cohort comparisons. This approach ensures a more robust and unbiased evaluation of differences within and between cohorts. We conducted the comparison of correlations across groups using the Wilcoxon rank-sum test.

## 3. Results and Discussions

In this section, we first investigated the hemodynamic distribution of the clinical data. Additionally, we quantified the chaotic components of the PPG using three sampling schemes and spectral decomposition. To determine the most suitable complexity parameters for estimating hemodynamics, we evaluated their correlation with the hemodynamic status and fluctuation. The causal effect between the chosen parameters and hemodynamic condition was investigated to further evaluate their relationship.

### 3.1. Hemodynamics Characteristics

**Figure 7:**
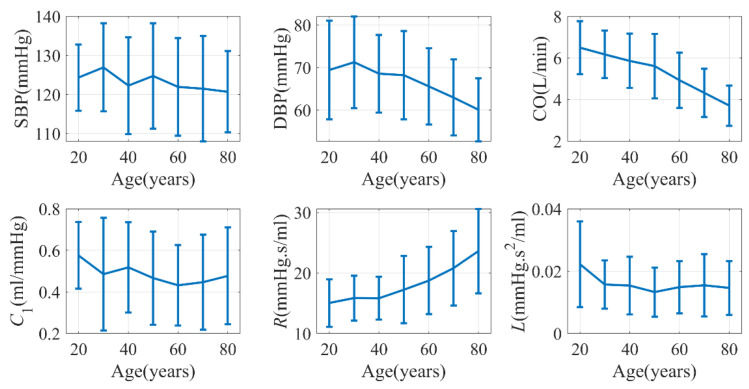
Age-dependence of hemodynamics characteristics (VitalDB). *C*_2_ was not shown due to its close relationship with *C*_1_.

A total of 652,313 windows were collected from the database, and the age stratified distribution of hemodynamic parameters is shown in Figure 7. The error bars in the figure represent the standard deviation of the respective hemodynamic parameters. Our analysis revealed several significant trends with age. DBP, CO, and *C*_1_ exhibited a significant decrease, whereas *R* showed a significant increase. On the other hand, SBP and *L* did not exhibit a significant trend with age.

Previous studies have demonstrated that CO may experience a decrease of 24% in males and 7% in females between the ages of 20 and 69 [51]. Additionally, *C*_1_ has been found to exhibit a negative correlation with age [52]. The change in peripheral resistance (*R*) can either increase or remain unchanged depending on the population under investigation [53]. Our findings aligned with these trends. However, it is generally known that SBP tends to increase with age [54]. Surprisingly, in our study, although PP—as the difference between SBP and DBP—significantly increased, SBP itself did not display a significant change with age. It is worth noting that the data in VitalDB predominantly comprise patients undergoing surgery, which may explain the inconsistency with the literature.

### 3.2. Quantification of the Chaotic Component of PPG Signals

The significance of the chaotic components is a noteworthy issue. As depicted in Figure 4, when the time resolution of the PPG measurement was one heartbeat, most of the long-term PPG signal (AC or DC fluctuation) exhibited fractal characteristics. However, as the time resolution decreased to 0.02 s (50 Hz), the percentage power of the fractal components was 4.0% in surgery patients and 13.2% in healthy subjects, on average. The difference between the two groups was statistically significant according to the Wilcoxon rank-sum test (*p* < 0.001). The percentage of chaotic components appeared to be relatively unaffected by hemodynamic status, such as CO (*r* = −0.04, *p* = 0.48). Nevertheless, we observed significant correlations between the percentage power of chaotic components and the standard deviation of DBP, *C*_1_, *R*, and *L*, with correlation coefficients of 0.16, 0.11, 0.23 and 0.13, respectively. It is noteworthy that SBP and CO were more influenced by active cardiac activity, whereas DBP, *C*_1_, *R* and *L* were more related to the passive vascular responses. If we consider the standard deviation as a measure of hemodynamic stability, our findings indicated that fractal components increase with greater hemodynamic instability. Furthermore, we assessed the impact of age on the percentage of chaotic power and found a marginally significant correlation (*r* = −0.10, *p* = 0.07) between the two variables. Weight and height had no influence.

The wavelet decomposition process proved to be effective in generating consistent chaotic components. Specifically, the correlation coefficient between the percentage power of the wavelet fractal components (8.23 Hz) and *C*_1_ exhibited a value of −0.43, as depicted in Table 3. The correlation displayed a monotonic decrease with wavelet frequencies, and there was a notable shift in the paradigm at 11.63 Hz. In Figure 5, it is evident that the oscillatory components vanished at 11.63 Hz, resulting in a shift in the paradigm. This finding suggests that incorporating fractal components is essential when assessing hemodynamics in the older population with stiff blood vessels. It is worth noting that the power of wavelet fractal components varies with hemodynamic status rather than its fluctuation, whereas the overall fractal power varies with hemodynamic fluctuations.

**Figure 8:**
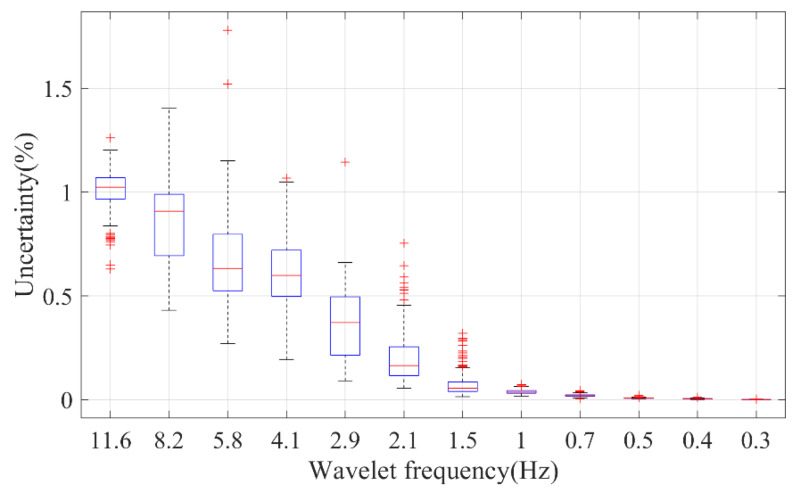
The fitting uncertainty of the HFD of different wavelet components of PPG. On each box, the central mark indicates the median, and the bottom and top edges of the box indicate the 25th and 75th percentiles, respectively. The whiskers extend to the most extreme data points not considered outliers, and the outliers are plotted individually using the ‘+’ marker symbol.

Another significant concern when estimating the fractal dimension is the associated uncertainty. The fractal spectrum, as depicted in Figure 4, may not adhere to standard characteristics. To quantify the fitting error, the variance–covariance matrix for the fitted coefficients was utilized [55]. Consequently, uncertainties were determined for various parameters. For example, the HFD of the PPG waveform, AC, and DC exhibited uncertainties of 2.2%, 11.3%, and 15.1%, respectively. As illustrated in Figure 8, the uncertainty for the HFD of the wavelet components is dependent on the spectral frequency, typically remaining below 1%. This minimal uncertainty in the HFD of wavelet components can likely be attributed to the simplistic frequency-dependent response of the cardiovascular system.

In summary, the PPG signal possesses fractal components that should be taken into consideration when dealing with individuals with stiff blood vessels or unstable hemodynamic conditions. The uncertainty in the HFD calculation depends on the sampling scheme, but it is generally low for most applications when using a window size of 100 cardiac cycles.

### 3.3. Correlation of PPG Complexities and Hemodynamic Status

We evaluated several commonly used complexity measures for their average correlation with main hemodynamic parameters: BP, CO, *C*_1_, and *R*. *C*_2_ and *L* were not displayed in the results due to their limited relevance to physiological applications in this context. We only presented complexity measures derived from the PPG raw signal, as it exhibited the strongest correlation with hemodynamics, as demonstrated in Supplementary Figure S1. Throughout the subsequent discussion, the term “complexity measure” without a subscript refers specifically to the complexity of the raw signal.

**Figure 9:**
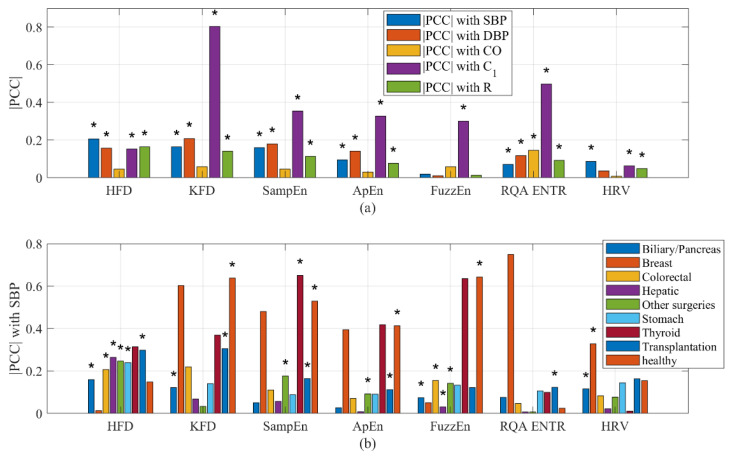
(**a**) Correlation of complexity measures with hemodynamics. (**b**) Correlation of complexity measures with SBP stratified by health conditions. PCC could be either positive or negative. * indicates a significant inter-subject (cohort) correlation (*p* < 0.05).

Among these measures, the HFD exhibited the strongest correlation with SBP, whereas the KFD displayed the strongest correlation with *C*_1_, as shown in Figure 9a. Morphological features typically exhibited a weak correlation with BP, CO, and *R*, but demonstrated a notably strong correlation with *C*_1_, as shown in Supplementary Figure S2. This observation was not unexpected, as it is widely accepted that PPG morphology reliably estimates blood vessel stiffness. It is important to note that our analysis is not exhaustive. We only included some commonly used complexity measures. It is possible that better performing complexity measures may exist.

A similar analysis was conducted on HRV. While HRV showed a weakly significant correlation with most hemodynamic parameters, the results revealed that HRV did not exhibit a significant overall correlation with CO. It is important to note that for certain individuals, the correlation between HRV and hemodynamics may be either significantly positive or negative. However, the statistical analysis revealed that the overall intersubject correlation between HRV and hemodynamics is considerably smaller. Likewise, the correlation between the HFD of wavelet components and BP was not found to be significant.

It was intriguing to observe that the type of surgery influenced the correlation between complexity measures and hemodynamics. For instance, the strongest average correlation observed between complexity features and SBP was 0.65 for SampEn (Thyroid). Following closely, the correlation between the KFD (Breast) and SBP reached −0.60. On the other hand, the weakest correlation was observed during biliary/pancreas surgeries, where the strongest correlation between any complexity measures and SBP reached only 0.15. It is important to emphasize that the accuracy of these intrasubject correlation estimation improves with longer recording times. As the recording time is usually longer than 1 h, an ample number of data points per subject are available, ensuring the robustness of the intrasubject correlation.

### 3.4. Sensitivity of Complexity Measures to Hemodynamics

The sensitivity of complexity measures to hemodynamics can vary depending on the hemodynamic fluctuation. For example, in an equilibrium system, the sensitivity of morphological features to BP is more influenced by the hemodynamic status [56], while in unstable hemodynamic conditions, the sensitivity of the FD to BP may rely more on the level of hemodynamic fluctuation. To describe this phenomenon, we employed Equations (2) and (3). “SBP” could be replaced by other hemodynamic parameters depending on the situation. Feature_m_ refers to morphological features, while FD refers to the HFD or KFD measures. *f*_m_ and *f*_d_ represent functions that establish a connection between the sensitivity of morphology and FD with respect to hemodynamics. The hemodynamic fluctuation was measured by calculating the standard deviation, denoted as σ.

(2) $$\frac{\partial {Feature}_{m}}{\partial SBP}=f_{m}(SBP,DBP,CO,C_{1},R)$$

(3) $$\frac{\partial FD}{\partial SBP}=f_{d}(\sigma (SBP),\sigma (DBP),\sigma (CO),\sigma (C_{1}),\sigma (R))$$

**Figure 10:**
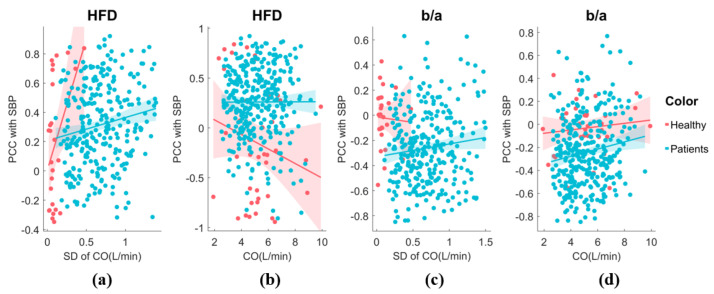
(**a**) The sensitivity of HFD to SBP exhibits variability in response to CO fluctuation. The analysis includes both healthy individuals and patients. A generalized linear model fit shows the 95% confidence interval (line + shaded area). (**b**) The relationship between the sensitivity of HFD to SBP and CO. (**c**) The relationship between the sensitivity of b/a to SBP and CO fluctuation. (**d**) The relationship between the sensitivity of b/a to SBP and CO.

To enhance clarity, we utilized SBP as an exemplar and conducted an examination of the sensitivity of the HFD to BP across varying hemodynamic fluctuation conditions. Additionally, we assessed the sensitivity of the morphological feature, b/a, to SBP for comparison purposes, as illustrated in Figure 10. Our analysis revealed that ∂HFD/∂SBP is primarily positive in surgery patients, also showing a positive correlation between ∂HFD/∂SBP and CO fluctuation. This suggests that the HFD becomes increasingly important in estimating SBP during unstable CO conditions. However, it is important to note that in healthy subjects, CO fluctuation was minimal during the 5 min test. While ∂HFD/∂SBP showed a change in signs with CO fluctuation, it was inconclusive and requires further investigation. On the other hand, ∂(b/a)/∂SBP was predominantly negative, with decreasing sensitivity to SBP as CO or CO fluctuation increases. b/a is known to correlate with instantaneous vascular compliance [37], which may explain its decreased sensitivity at high CO.

**Figure 11:**
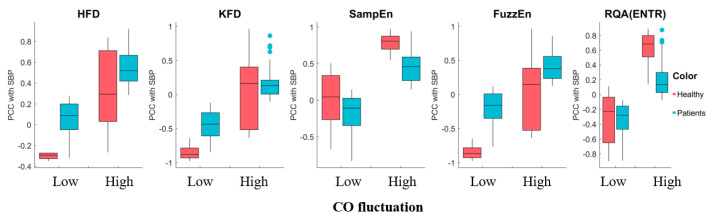
The sensitivity of complexity measures to SBP exhibited variability in response to high and low CO fluctuation. The central mark represents the median, while the bottom and top edges of the box represent the 25th and 75th percentiles, respectively. The whiskers extend to the farthest data points that are not considered outliers, and outliers are individually marked.

Most of these complexity feature sensitivities displayed a significant trend depending on CO variation, indicating that variations in hemodynamics can result in different temporal patterns, as shown in Figure 11. SampEn exhibited consistent increases in sensitivity to SBP with higher CO fluctuations. On the other hand, HFD and FuzzEn demonstrated increased sensitivity to SBP with higher CO fluctuations, but only in surgery patients. RQA(ENTR) showed increased sensitivity only for healthy subjects. Interestingly, the KFD displayed decreasing sensitivity, indicated by lower |PCC| values, suggesting a different origin compared to the HFD. We categorized the CO fluctuation as either “low fluctuations” or “higher fluctuations” by dividing it with the median value. The morphological features, SPMEAN and SPVAR, exhibited similar performances to b/a, while other features generally showed insignificant changes in sensitivity with CO fluctuation. We have shown selected features in the Supplementary Figure S3.

### 3.5. Granger’s Causality Test

This analysis encompassed a total of 401 subjects, and we explored both unidirectional causality and mutual feedback, as presented in Table 4. Among the subjects examined, 195 individuals showed unidirectional hemodynamics caused HFD, while feedback was observed in 136 subjects, indicating that the HFD also influences the hemodynamics in these cases. Only 70 subjects exhibited no discernible causal relationship. The HFD offers a more precise mathematical estimation of the FD, whereas the KFD [42,43], although it may display a good correlation with hemodynamics in specific scenarios, lacks a strong causal relationship with hemodynamics. This can potentially be attributed to the calculation method of the KFD, which tracks the vessel wall movement trajectory [40,43].

A Wilcoxon’s rank-sum test with uneven numbers of subjects showed that, for subjects exhibiting feedback, there was a significant increase in σ_CO_ (*p* = 0.04). For instance, if the HFD displayed bidirectional feedback with hemodynamics, the median σ_CO_ was 0.76 L/min, whereas for subjects with no feedback, the median σ_CO_ was 0.61 L/min. This finding suggests that in subjects without feedback, one potential explanation could be that the absence of sufficient disturbance fails to trigger the activation of fractal behavior.

Despite achieving a high correlation with *C*_1_, there was a much smaller causal relationship between the KFD and hemodynamics. Combined with the finding that the KFD is more sensitive to DBP, this suggests that the KFD might be more associated with structural fractality instead of hemodynamic responses. Notably, we observed that HRV predominantly acted as the influencer, rather than being influenced, implying that HRV is an active stimulus. Similarly, b/a was more of an influencer, which was also heavily influenced. The interaction time was also short, so that mutual feedback was less. These noteworthy outcomes contribute valuable insights into the intricate interplay between hemodynamics and the HFD within our study population.

In conclusion, our analysis suggests that elevated BP and fluctuations in hemodynamics are likely the main contributors to the observed fractal behaviors. Additionally, it is possible that the structural fractality of vasculature may also contribute to the occurrence of such behaviors.

### 3.6. Potential Improvement by Adding Temporal Complexity Information

While not explicitly utilized, certain studies may employ the temporal association of data or spectral information to enhance the estimation of hemodynamics. Among these, the most prevalent topic is the estimation of BP. We used the single-site finger PPG-derived BP as an example, as shown in Table 5. Although the methods employed in these studies vary, a noticeable trend of improvement can be observed when incorporating temporal information [2,57,58], spectral information [59], stage-wise hemodynamic information, such as SV and CO [60], and bidirectional temporal information [61]. It is important to note that the specific results obtained may be influenced by factors, such as the data source, validation process, and the utilization of the black box of deep neural networks. Nevertheless, it is evident that the inclusion of temporal information contributes valuable insights to the estimation of hemodynamics.

## 4. Discussion

### 4.1. Novelty of the Study

Measuring ambulatory vital signs with wearable devices has always been a challenge. In fact, even when individuals are immobile, various factors like breathing and neurological activities contribute to fluctuations in the cardiovascular system. This lack of true equilibrium in the cardiovascular system introduces hysteresis, potentially affecting the accuracy of measurements. Machine learning algorithms have shown promising results in overcoming these challenges. However, understanding the exact mechanism behind their improved performance remains difficult.

In our previous study [18], we observed temporal complexities due to hemodynamic fluctuations but lacked a clear understanding of their nature. Our current study used the IRASA algorithm to confirm the fractal nature of PPG’s temporal patterns and quantify their contribution.

Key findings include:

(1) Multiscale fractal components’ power increased with declining vascular compliance, indicating their significance in individuals with stiff blood vessels.
(2) The HFD and KFD had the strongest correlation with SBP and DBP, respectively, compared to other complexity measures.
(3) The sensitivity of the FD to BP was more pronounced in individuals with higher hemodynamic instability.
(4) The Granger causality showed that both feedback and unidirectional relationships exist between hemodynamics and HFD, while HRV was the influencer rather than being influenced. The KFD may be more relevant to vascular structural complexities.
(5) Subjects with clear causal feedback between HFD and hemodynamics exhibited significantly increased fluctuations in cardiac output.
(6) Healthy subjects displayed a greater occurrence of fractal components, which should be taken into consideration when estimating hemodynamic parameters. Additionally, less invasive surgeries, particularly those that do not significantly affect the cardiovascular system, showed a relatively stronger complexity–hemodynamics correlation.

To our knowledge, this study is the first attempt to clearly establish a connection between hemodynamic condition and fractal properties of PPG signals. The results of our study can serve as a valuable starting point for researchers seeking a suitable fractal description of the system and an explanation for the observed phenomena.

### 4.2. Comparison with Previous Studies

Although previous studies have confirmed that the PPG signal is fractal [15,16], very few studies have linked the FD to hemodynamics [62], and most of them are empirical without a comparison to other complexity measures. [63,64]. In this study, we quantified the fractal components and tested the potential importance of different sampling schemes, such as wavelet decomposition, by sampling per cardiac cycle and at 50 Hz. Our study revealed a strong correlation between the FD and BP, indicating a high association between these variables. When examining the wavelet decomposition, we observed an increase in the power of fractal components in relation to vascular stiffness. We also showed that FD is a better indicator for hemodynamic estimation, compared to other popular complexity measures.

While HRV has been extensively studied as a dynamic feature, its influence on improving blood pressure primarily pertains to assessing neurological activity [65,66,67]. It represents the “active” cardiac activities and does not encompass all aspects of the dynamic process. Our causal analysis aligned with this physiological explanation. Additionally, our findings indicated that chaotic behavior is largely caused by fluctuations in hemodynamics, which occur as a passive vascular response. The presence of feedback suggests a closed-loop nature within the cardiovascular system. Comparing these findings with HRV may provide valuable insights into the physiological source of complexities observed in PPG.

### 4.3. Limitations of the Study

In our study, the database primarily comprised surgery subjects. While we did include healthy subjects in the cohort, it is important to note that their measurement duration was shorter, and their hemodynamic fluctuation was significantly lower compared to the surgery patients. To obtain more comprehensive results, the same procedure should be tested on datasets that encompass a broader range of subjects. Although the influence of sex on the correlation between PPG and hemodynamic is unlikely, further exploration is warranted to better understand its role. Lastly, we employed estimated hemodynamic parameters to produce the relationship between the FD of PPG and hemodynamics. It is imperative to validate these findings through medical ultrasound and total peripheral resistance (TPR) measurements.

While the WK4 model and the fractality of PPG share some resemblances to Rössler’s chaos, it is important to note their distinct structures. Therefore, additional research efforts should be dedicated to unraveling the nature of the attractor.

### 4.4. Suggestions for Future Work

The cardiovascular system is a closed-loop system. Through causal analysis of experimental data, we identified both unidirectional and feedback interactions between hemodynamic fluctuation and temporal fractality. To gain a deeper understanding of this interaction, future research should involve in silico simulations using closed-loop models. Additionally, there are unresolved issues that need to be addressed. Quantitative analysis of the fractal components is necessary to determine the specific contributions from the vascular tree, cardiac pacing, and vascular hysteresis. Furthermore, conducting further investigations on the age effect will enhance hemodynamic estimation in different age groups. Lastly, it is crucial to determine the exact nature of the fractal attractor by expanding our knowledge in this area.

## 5. Conclusions

In this study, we systematically analyzed the fractal behavior of PPG and discovered a clear dependence of the FD on hemodynamic conditions. This study may help unravel the origin of fractality and result in better machine learning algorithms.

## Figures and Tables

**Table 1:** Biometric and BP distribution.

	VitalDB		Healthy	
	Mean ± STD	Range	Mean ± STD	Range
Age (years)	55.5 ± 14.9	20–82	33.9 ± 14.5	18–65
Height (cm)	163.9 ± 8.4	147–183	171.1 ± 10.7	148–198
Weight (kg)	62.2 ± 11.1	41–89	76.0 ± 17.6	48–136
SBP (mmHg)	122.3 ± 18.6	84–168	146 ± 25	80–193
DBP (mmHg)	66.1 ± 12.8	41–98	88 ± 18	43–109

**Table 2:** Definition of selected morphological features.

Features	Definition
AC	Pulsating amplitude of PPG [33]
DC	Mean of PPG baseline [33]
AREA	Area under the normalized PPG waveform [34]
SPMEAN	Mean upstroke slope during the systolic period [35]
SPVAR	Variation of upstroke slope (standard deviation) during the systolic period [36]
DPMEAN	Mean downstroke slope during the diastolic period [35]
DPVAR	Variation of downstroke slope (standard deviation) during the diastolic period [35]
b/a	Height of the early negative peak over the early positive peak of second derivatives of PPG [37]

**Table 3:** The Pearson correlation coefficient (PCC) between the percentage power of wavelet fractal components and *C*_1_.

	Correlation between the Percentage Power of Wavelet Fractal Components and *C*_1_						
Wavelet frequency (Hz)	11.63	8.23	5.82	4.11	2.91	2.06	1.45
PCC	0.01	−0.44 *	−0.34 *	−0.32 *	−0.21	−0.19	−0.13

* significant correlation (*p* < 0.05).

**Table 4:** Number of subjects with certain causal relationships.

	HFD	KFD	LF/HF	AC	b/a
**Unidirectional** **hemodynamics-caused complexity**	195	74	23	95	192
**Unidirectional** **complexity caused** **hemodynamic changes**	62	60	276	151	35
**Bidirectional feedback**	74	54	20	82	115
**No causality**	70	213	82	73	59

**Table 5:** Comparison with previous studies.

Studies	Algorithm	MAE (mmHg)		Data Source
		SBP	DBP	
**Radha et al.** [2]	LSTM	8.22 (RMSE)	6.55 (RMSE)	106 healthy subjects
**Harfiya et al.** [57]	LSTM	4.05	2.41	MIMIC dataset
**Slapničar et al.** [59]	Spectro-Temporal Deep Neural Network	9.43	6.88	MIMIC dataset
**Wang et al.** [58]	LASSO-LSTM	4.95 6.14	3.15 5.61	UCI-ML-BP; MPC-FAHUSTC dataset
**Atef et al.** [60]	LSTM with multistage transfer learning	2.03	1.18	MIMIC dataset
**Meng et al.** [61]	DC-Bi-RNN	3.21	1.80	MIMIC dataset

RMSE: Root Mean Square Error; MAE: Mean Absolute Error; LSTM: Long Short-Term Memory; DC-Bi-RNN: deep convolutional bidirectional recurrent neural network; MIMIC: Medical Information Mart for Intensive Care.

## Data Availability

The datasets utilized in this study are accessible at the official website of VitalDB, located at (https://vitaldb.net/). The researchers accessed the database between July and October of 2023.

## Supplementary Materials

The following supporting information can be downloaded at: https://www.mdpi.com/article/10.3390/e25121582/s1, Figure S1: Correlation of complexity measures with hemodynamics; Figure S2: Correlation of morphological measures with hemodynamics, stratified by health conditions; Figure S3: The sensitivity of morphological features to SBP exhibits variability in response to CO fluctuation.
