# Supplementary material for: Analysis of the Chaotic Component of Photoplethysmography and Its Association with Hemodynamic Parameters
Source: Entropy (Basel). 2023 Nov 24;25(12):1582. doi: 10.3390/e25121582 (PMC10742563; doi:10.3390/e25121582)
Supplement: Supplementary file 1 [file entropy-25-01582-s001.zip › entropy-2622939-supplementary.pdf]

Analysis of the Chaotic Component of Photoplethysmography and Its Association with Hemodynamic Parameters: Supplementary material

Xiaoman Xing [1,2,*], Wen-Fei Dong [2,*], Renjie Xiao [3], Mingxuan Song [4] and Chenyu Jiang [5]

1 School of Biomedical Engineering (Suzhou), Division of Life Sciences and Medicine, University of Sciences and Technology of China, Suzhou 215163, China
2 Suzhou Institute of Biomedical Engineering and Technology, Chinese Academy of Sciences, Suzhou 215163, China
3 Medical Health Information Center, Suzhou Institute of Biomedical Engineering and Technology, Chinese Academy of Sciences, Suzhou 215163, China
4 Suzhou GK Medtech Science and Technology Development (Group) Co. Ltd, Suzhou 215163, China
5 Jinan Guoke Medical Technology Development Co., Ltd., Jinan 250100, China
* Correspondence: xingxm@sibet.ac.cn (X.X.); wenfeidong@126.com (W.-F.D.)

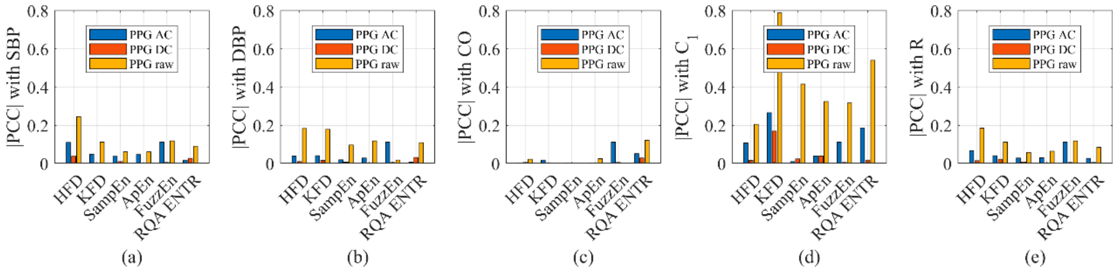

Supplementary Figure S1. Correlation of complexity measures with hemodynamics.

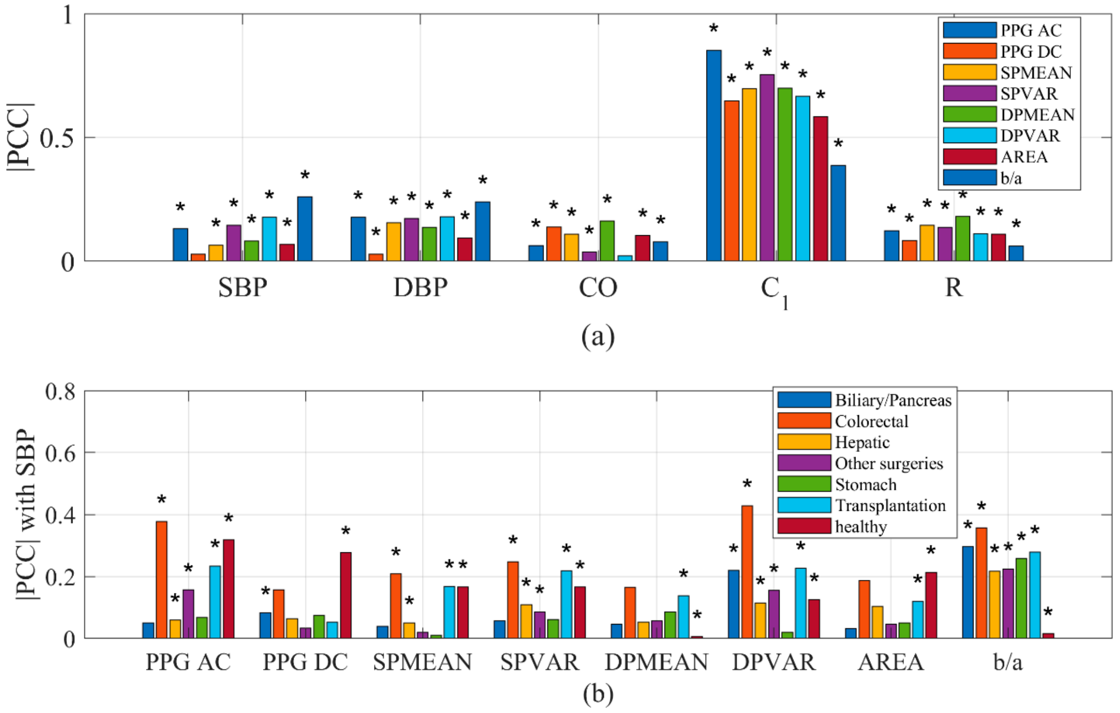

Supplementary Figure S2. (**a**) Correlation of morphological measures with hemodynamics. (**b**) Correlation of morphological measures with SBP stratified by health conditions.

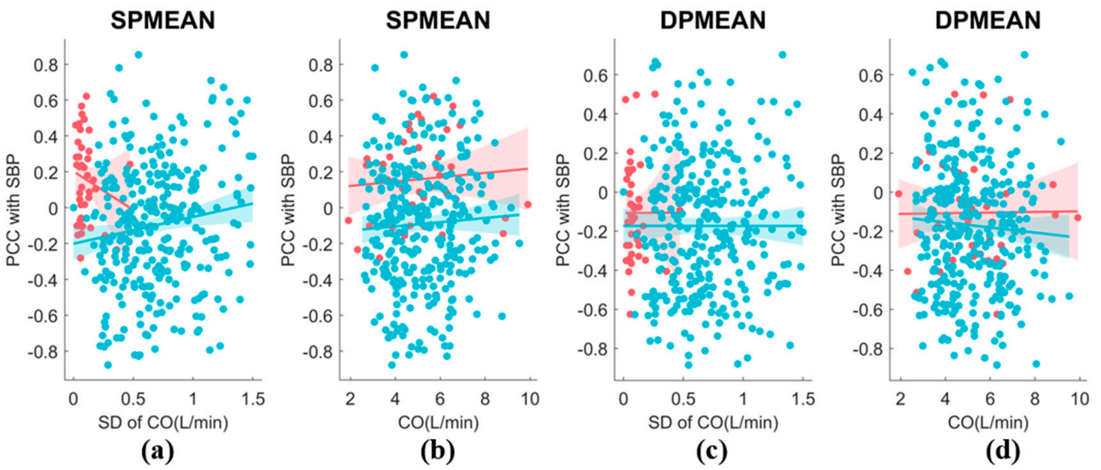

Supplementary Figure S3. (**a**) The sensitivity of SPMEAN to SBP exhibits variability in response to CO fluctuation. A generalized linear model fit shows the 95% confidence interval (line+shaded area). (**b**) The relationship between the sensitivity of SPMEAN to SBP and CO (**c**) The relationship between the sensitivity of DPMEAN to SBP and CO fluctuation. (**d**) The relationship between the sensitivity of DPMEAN to SBP and CO.
